# Music listening for psychological well-being in adults with acquired vision impairment: a feasibility randomised controlled trial

**DOI:** 10.3389/fpsyt.2025.1505283

**Published:** 2025-02-25

**Authors:** Nurbanu Somani, Alexander Street, Eldre Wiida Beukes, Jufen Zhang, Peter M. Allen

**Affiliations:** ^1^ Vision and Hearing Sciences Research Centre, Anglia Ruskin University, Cambridge, United Kingdom; ^2^ Cambridge Institute for Music Therapy Research, Anglia Ruskin University, Cambridge, United Kingdom; ^3^ School of Medicine, Faculty of Health, Education, Medicine and Social Care, Anglia Ruskin University, Chelmsford, United Kingdom

**Keywords:** vision impairment, music-based interventions, mindful music listening, psychological well-being, feasibility randomised controlled trial, self-care music intervention

## Abstract

**Purpose:**

Vision impairment (VI) has a profound impact on mental health and well-being. Music-based interventions, such as active music listening, have potential to induce relaxation, improve mood, and reduce stress. This study investigated the feasibility and acceptability of a supportive self-care music intervention in adults with acquired VI, who ran their listening program independently.

**Methods:**

A two-arm, parallel-group, single-blind feasibility randomised controlled trial compared: (1) daily music listening with brief mindfulness instructions and (2) daily music listening alone. The study adhered to the CONSORT extension for pilot and feasibility trials and the music reporting checklist. Feasibility was determined by collecting data on attrition, through recruitment and retention at pre-post and 3-month follow-up, including adherence and fidelity which were determined by participants’ questionnaire and daily log completion, respectively. Acceptability was determined through data capture in the questionnaires. To assess whether the music intervention had an impact on anxiety and depression and stress, the Hospital Anxiety Depression Scale (HADS) and the Perceived Stress Scale (PSS-14) were used.

**Results:**

Eighty-one VI participants were randomised to mindful music listening (n = 41) or music listening alone (n = 40), with 85% (n = 69) completing the intervention (post intervention end-point). A rating of ‘highly satisfied’ was given for the intervention by 77% (*n* = 53) of participants. Feasibility was determined through retention which was 71% at the three-month follow-up, and adherence to the daily log completion, which was low (16%), resulting in insufficient data to assess fidelity to the music listening. HADS and PSS-14 data indicated a reduction in anxiety, depression and stress at post-intervention phase.

**Conclusions:**

The recruitment and retention rates suggest that an efficacy study is feasible. However, adherence to daily log completion was low, resulting in uncertainty regarding the fidelity of the listening conditions. While completing the daily logs was not required in this study, it should be mandatory in future trials.

**Clinical Trial Registration:**

https://clinicaltrials.gov/study/, identifier NCT05243732.

## 1 Introduction

A vision impairment (VI) significantly influences multiple facets of an individual’s life and is correlated with diminished functional capacity (1). The specific impact of VI may vary according to the severity of vision loss, but frequently encompasses challenges related to reading, writing, interpreting non-verbal cues (2–4) and participating in social interactions (5–8). These challenges can have adverse effects on a person’s mental health, contributing to conditions such as depression (5, 6, 9, 10), anxiety (10–13), emotional distress (6, 7, 14), feelings of loneliness (15–17), social isolation (16), and a diminished sense of belonging (7, 16, 17). In addressing the challenges posed by VI, it is important to prioritise the enhancement of well-being among affected individuals, recognising the interplay of psychological, physiological, and social factors that collectively influence mental health and psychosocial well-being (17, 18).

The concept of well-being is multifaceted and lacks a singular, universally accepted definition. Nevertheless, there exists a general consensus that well-being encompasses the presence of positive emotions and moods (e.g., contentment, happiness), the absence of negative emotions (e.g., depression, anxiety), life satisfaction, positive functioning, and a sense of health and vitality (19). Researchers from various disciplines may employ the term “well-being” in accordance with their specific field of interest, which can encompass physical, social, developmental, activity-based, emotional, psychological, life satisfaction, domain-specific satisfaction, engaging activities, and work-related dimensions (19, 20).

Effective strategies for promoting well-being among individuals with VI may involve physical activity (21), engagement in the arts (22), and mindfulness practices (23). Additionally, music-based interventions represent a promising approach for enhancing well-being in this population (24). Studies on music interventions demonstrate that active music listening, consciously focusing on music rather than passively listening in the background may serve as a distraction from unpleasant thoughts and feelings (25, 26), induce relaxation, positive mood changes, and evoke memories (26–28). The broaden-and-build theory (29) supports this idea by proposing that experiences of positive emotions, such as those elicited by music, broaden individuals’ “momentary thought-action repertoires.” This broadening effect can contribute to long-term improvements in psychological and physical well-being (29).

Music, as one of the most powerful triggers of emotions, can function as a catalyst for fostering positive emotional experiences (25–28), consequently offering potential relief from feelings of depression, anxiety, and stress. This aligns with components of Seligman’s PERMA model (Positive Emotion, Engagement, Relationships, Meaning, and Accomplishments.) (30), emphasising positive emotions as a crucial element of overall well-being. Music can be used in a variety of ways to promote well-being, especially in individuals with VI (31). Linnemann et al. state that listening to music is an easy, accessible, and cost-effective strategy to incorporate into one’s self-care routine (32). This idea may be derived from Orem’s (1971) theory of self-care – that certain activities/techniques can be used by individuals to take care of their own physical, psychological, cognitive and/or emotional well-being (33). Music listening can be considered as a self-care technique (33) in that it is a voluntary activity to improve well-being.

Presently, there is a limited number of self-care music-based interventions that have been investigated remotely in general populations (34–39). However, the ones that have been explored demonstrate successful implementation, requiring minimal supervision with initial instructions provided by researchers. This is crucial as self-care is associated with lower healthcare utilisation, resulting in fewer visits to healthcare providers or facilities, contributing to more efficient and cost-effective healthcare practices (40).

To advance knowledge in this area of research, a feasibility and acceptability randomised controlled trial (RCT) was conducted exploring a participant delivered self-care music intervention for adults with acquired VI, aimed at promoting psychological well-being. The rationale for this research was grounded in a deliberate focus on acquired VI, distinguishing it from conditions like congenital or surgically rectifiable VI, such as cataracts. The choice is informed by the substantial impact on mental well-being caused by acquired VI (41, 42).

### 1.1 Aims and outcome measures

The overall aim was to explore whether, in a randomised controlled trial (RCT) context, individuals with acquired VI could self-administer daily music listening (either alone or with mindful music instructions) for well-being within their homes over a four-week period.

#### 1.1.1 Primary aims and outcome measures

- Intervention Feasibility will be reported as the percentage of participants who enrol and complete the entire study.
  - Attrition rate of the intervention will be determined by the percentage of recruited participants remaining at the intervention end point.
  - Adherence to completion of the music listening diary log will be reported as the percentage of the participants who complete the diary log.
  - Treatment adherence will be reported as the percentage of the participants who complete all aspects of the study.
  - The acceptability and accessibility of the intervention will be reported as the rating of and percentage of participants who evaluated the intervention.

#### 1.1.2 Secondary aims and outcomes measures

- Changes in Depression, Anxiety and Stress levels will be self-rated by participants using the Hospital Anxiety and Depression Scale (HADS) and the Perceived Stress Scale (PSS-14).

## 2 Materials and methods

### 2.1 Trial Design

**Figure 1:**
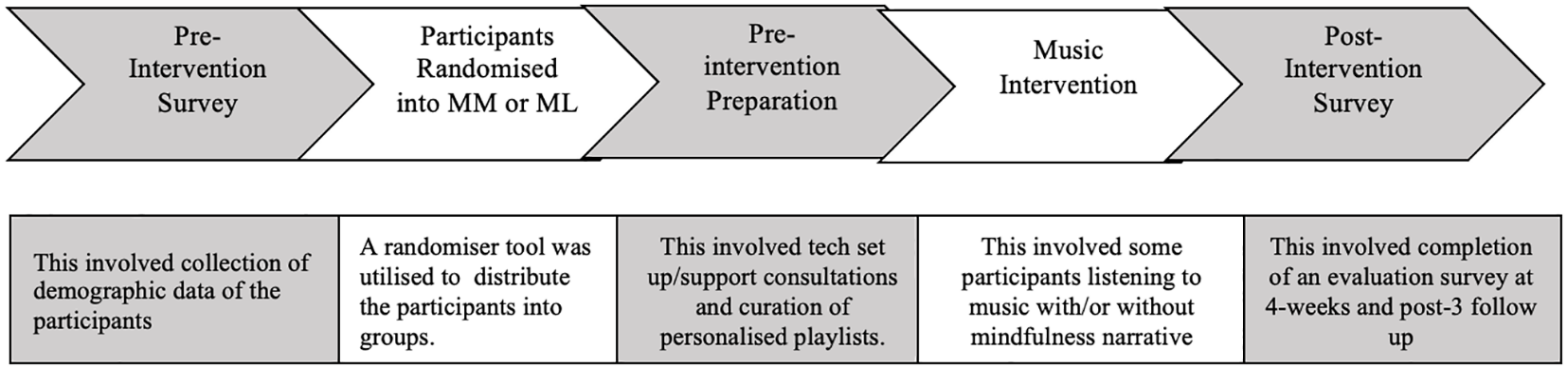
The workflow of the intervention, including the pre-intervention survey to the end of the intervention itself.

A two-arm, parallel group, single-blind feasibility randomised control trial (RCT) comparing (1) mindfulness music listening (MML) and (2) music listening alone (ML) for four-weeks in participants’ homes. Data were collected remotely on efficacy in reducing symptoms of anxiety and depression, and treatment fidelity. During the study, all participants continued their daily routines as usual. **Figure 1** shows participant flow through the study. Reporting follows the CONSORT extension for pilot and feasibility trials guidelines (43). The study was approved by the Faculty Research Ethics Panel at Anglia Ruskin University (FREP 1021-07) and conformed to the tenets of the Declaration of Helsinki. All participants gave written informed consent online. The protocol was registered on clinicaltrials.gov with identification number (NCT05243732).

#### 2.1.1 Feasibility assessment

**Figure 2:**
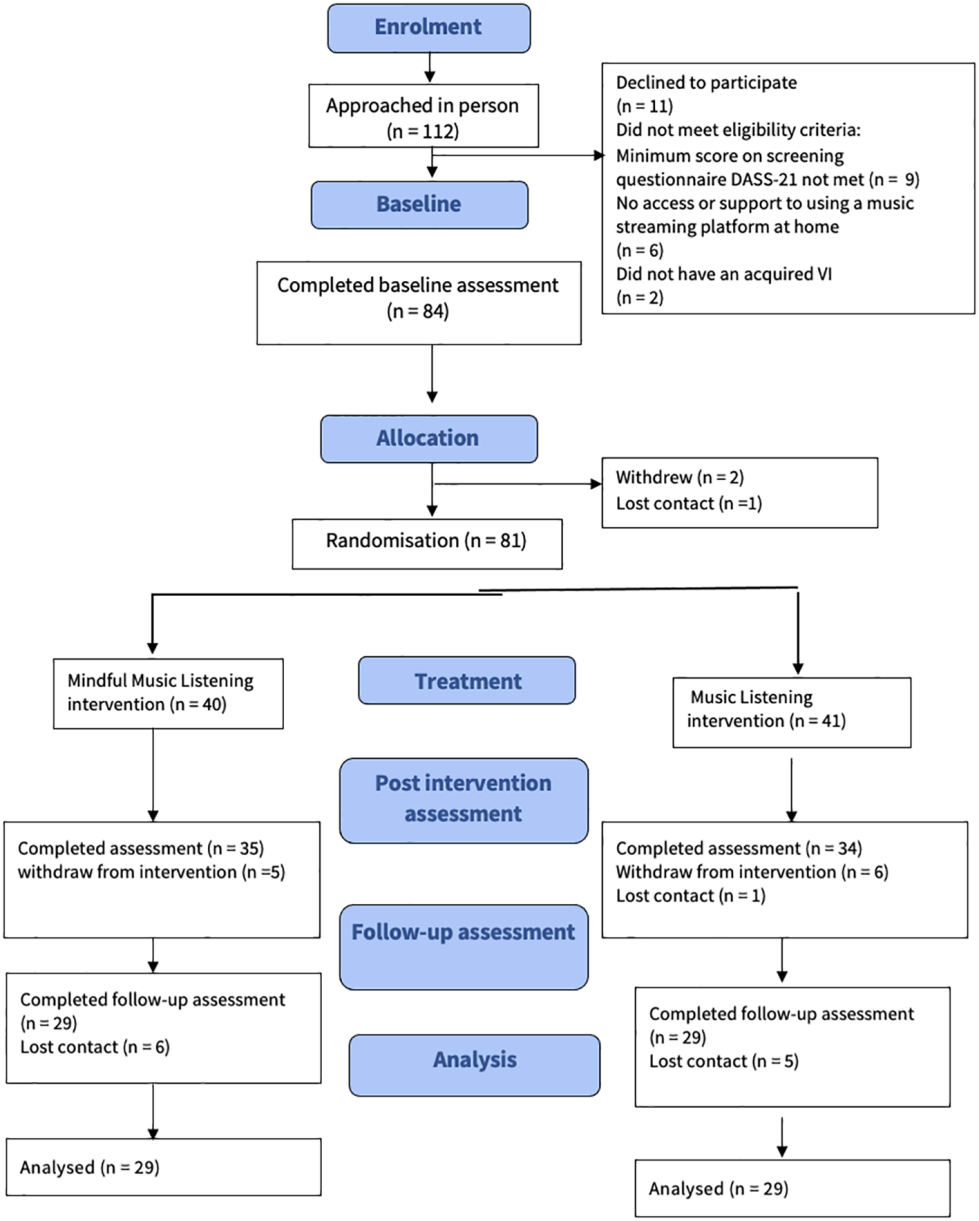
The CONSORT flow diagram.

To determine feasibility for this study, data were collected on recruitment, retention, adherence, and reasons for withdrawal. These data are presented in the CONSORT flow diagram (**Figure 2**). Adherence and fidelity to listening exercises were recorded via daily logs during the four-week active intervention period.

### 2.2 Participants

Eligibility Criteria.

- Adults over 18 years with an acquired visual impairment (VI) (classified as Sight Impaired (SI) if their visual acuity is between 3/60 and 6/60, or if they have a visual acuity of 6/60 or above but with a significantly reduced field of vision or Severely Sight Impaired (SSI) individuals have a visual acuity of less than 3/60 with a full visual field, or a visual acuity between 3/60 and 6/60 with a severe reduction in the field of vision. These classifications are based on guidelines provided by Royal National Institute of Blind People (RNIB) (44), ‘3/60’ refers to a visual acuity measurement assessed using the Snellen chart, where the individual can see at 3 meters what a person with normal vision can see at 60 meters (45).
- Have symptoms of anxiety and/or depression, assessed via the Depression, Anxiety and Stress Scale (DASS-21) with a depression score of 10 or higher, and/or anxiety score of 8 or higher, and/or stress score of 15 or higher (46).
- Able to use and/or subscribe to a streaming provider to listen to music online.
- Own a smartphone/tablet or technology that supports their music streaming platform.
- Have capacity to consent.

Exclusion criteria.

- Hearing impairment that renders the individual unable to listen to music and follow a conversation on the telephone.
- If the DASS-21 scores are below the specified threshold for depression (score: 9 or less) and anxiety (score: 7 or less) and stress (score: 14 or less) (46).

Sample size estimation: *A priori* sample size estimation for using repeated measures, between factors ANOVA (analysis of variance) to test the means of three measurements for the same test subjects for differences was calculated using GPower v.3.1 (47). Setting alpha at 5% and beta at 95% and effect size of d=0.545, which was identified in the most recent meta-analysis and literature review on music interventions looking at psychological stress-related outcomes (48). It was found that 32 participants per arm in total would be required. However, to account for attrition, 39 participants per arm, assuming a 20% refusal or drop-out rate of the actual sample calculated (47) was the target recruitment.

#### 2.2.1 Recruitment

Participants were recruited over an eight-month period in 2022, from ten sight loss charities and higher education institutions across Cambridgeshire, Essex, Hertfordshire, and Greater London. Recruitment was conducted through advertisements via email mailing lists, newsletters, social media platforms including Facebook groups and face-to-face visits to various social gatherings organised by charities and residential care or nursing homes. In addition, snow balling sampling technique such as word-of-mouth were also utilised. Participants received no financial incentive for their participation in the study.

#### 2.2.2 Randomisation and stratification

Following collection of baseline data, eligible participants were randomised to the mindful music listening (MML) or music listening (ML) groups in a 1:1 ratio, based on an allocation sequence generated by software (https://www.randomizer.org/) and block randomisation to ensure equal distribution between groups (stratification) (49). Stratification was based on (i) DASS-21 scores, (ii) number of years living with a VI, and (iii) age. Due to the exploratory nature of this research, the researcher (NS) was managing the intervention alone and was therefore aware of the group assignments. Participants were aware of their allocated group as they would naturally recognise whether they were listening to preferred music only or music combined with mindfulness. However, to minimise the risk of bias, participants were not informed of the study hypothesis or its underlying aims.

#### 2.2.3 Compilation of personalised playlists

As part of the pre-intervention survey, participants were asked to complete a music preference section. This section was adapted from the Assessment of Personal Music Preference (APMP) devised by Gerdner et al. (2000) (50). It included questions relating to participants’ musical background and their preferred genres, composers, and performers. When a participant’s music preference section was incomplete or not sufficiently detailed to compile a suitable playlist, the researcher (NS) contacted those individuals to book a one-to-one telephone or video consultation to help them identify their preferred music. The length of the consultation was up to an hour and participants were offered a maximum of two consultations where necessary. For example, if the participant stated they like pop music but did not specify particular artists or songs, the researcher played samples of different types of pop music by using “share” audio function on the video conferencing software or loudspeaker function on the telephone to help them narrow down the most relaxing and uplifting pop music. This was also applied to other music genres. Thereafter, a playlist was generated on the individual’s preferred music streaming platform utilising their artificial intelligence (AI) to tailor song recommendations. The music streaming platforms analyse song search history, considering factors such as genre, artist, mood, and user behaviour, enhancing personalisation and facilitating music discovery. This method was utilised for all participants to ensure a preferred playlist was generated in a time efficient manner. This was conducted in line with the literature that suggests that song recommendations using this method is an appropriate way to generate personalised, meaningful playlists and exposure to new songs based on their preference (51).

#### 2.2.4 Technical training

Technical training and support were provided to all study participants and/or carers supporting them as part of the intervention. All participants received an initial one-to-one training session over Zoom/Teams or telephone, for up to an hour, where the researcher talked through navigating their preferred music streaming platform, how to listen, save and/or recover deleted files and/or download the emailed playlist onto their preferred platform application (e.g., Spotify, Apple Music) or their smart device (smartphone, tablet/iPad, Alexa/Google Play/Amazon Echo) and opening an account on a music streaming platform if they did not have one. In addition, participants were asked to confirm they were able to use the music streaming platform, check the sound quality of their device speakers/headphones was optimal, confirmed they understood the intervention exercise they were allocated and raise any further questions they had. Some training sessions included screen share, so the participant/carers could be shown the actual screens they need to access. Participants were also sent an email with the same information.

During the intervention period, ongoing technical support was also provided on request. If at any point the participants had difficulties using the music streaming platforms they emailed the researcher, who would work with them to find a solution.

### 2.3 Interventions

Participants were instructed to self-administer the intervention for a minimum of 30 minutes and up to 1-hour in a single sitting per day, any time of the day that was convenient for the participant, using their personal smart device (smart phone, tablet, Alexa, Google Play, Amazon Echo, laptop, computer), five days a week, for four weeks. Participants were recommended to listen to their preferred music playlist at home in an area that was quiet, where they would not be disturbed or distracted, enabling them to fully focus on the music. Participants were also asked to keep an optional daily log of listening, to inform on adherence to the interventions.

#### 2.3.1 Mindful music condition

Participants who were allocated the mindful music condition were emailed a brief mindful music exercise to complete daily prior to listening to their preferred music playlists. This focused on paying attention to the present moment and followed published mindfulness guidelines (52). For example, if participants were to notice any thoughts or sensations arising either during the brief exercise or during subsequent music listening, they were told to allow them to pass and to gently bring their attention back to the exercise/music.

#### 2.3.2 Music listening condition

No specific listening instructions were given to the music listening-only condition, other than listening to their music at home in a quiet area.

### 2.4 Data collection

#### 2.4.1 Baseline and post-intervention survey

The baseline survey collected data on demographics, ocular history, lifestyle factors, music preferences, medical history and used psychological assessments. The post intervention survey asked participants to rate their experiences with the interventions and complete the post intervention psychological assessments.

#### 2.4.2 Fidelity and adherence

To monitor treatment fidelity and adherence during the intervention sessions, participants were requested to complete a daily log of the music listening sessions via Qualtrics or print out the Microsoft word document of the daily log and hand write their responses and post back to the researchers. The daily log requested information on: if they listened with another person; what time of day they listen to the music; the duration of the music listening; their mood/feelings before and after music listening; relaxation level before and after music listening; how they listened to music (e.g., headphones or speakers or integrated); if there was a change in their daily routine; if there were any other factors that they felt may have influenced their experience of the music listening that day; if they moved along to the music; if they listened to the music at home, somewhere quiet; how easy it was to access the music playlists; to rate the quality of the sound; if they needed the mindfulness narrative recording or if they learnt how to do it without and if so at which point in the study. All items on the log were based on the TIDieR checklist (53) and tailored accordingly for this study (see **Supplementary Data Sheet 2**).

#### 2.4.3 Intervention feasibility, acceptability and accessibility

Intervention feasibility was assessed via completion rates of the post intervention survey and acceptability was rated on 5-point and 10-point Likert scales; for example, where 5 indicated very accessible and 10 indicated highly satisfied/most enjoyable whereas 1 indicating not accessible or not satisfied. Additionally, mood ratings were recorded in the daily logs, using a 5-point Likert scale, showed either a positive change in mood (e.g., from 1 = sad, 2 = down, 3 = neutral, 4 = calm 5 = happy) before and after the music sessions.

#### 2.4.4 Psychological assessments

All psychological outcomes used were well-established instruments that are widely used in the VI populations, vulnerable populations and older adults (54–62). All outcome assessments were self-reported and administered online to minimise researcher-participant response bias. To accommodate the varying severities of VI, Qualtrics was selected as the survey host due to its compatibility with assistive technologies commonly used by individuals with VI. Accessible formats were incorporated, including screen reader compatibility, adjustable font sizes, and high-contrast layouts, ensuring that participants could independently engage with assessment.

For the intervention eligibility screening of depression, anxiety, and stress DASS-21 (46) was used. The Hospital Anxiety and Depression Scale (HADS) (56) and Perceived Stress Scale (PSS-14) (57) were selected as the primary outcome measures for mental health.

#### 2.4.5 Follow-up

Follow-up assessments on mental health, stress and intervention evaluation survey were completed immediately post-intervention, at 4-weeks post intervention, and after 3-months.

### 2.5 Data analysis

All quantitative data analyses were performed using IBM SPSS Statistics version 26.0 (IBM Corp, 2019). Regarding the psychological assessments, participants who did not complete the full intervention and had missing data, were not used in the data set that was analysed. Categorical variables were summarised using frequency counts and percentages. Continuous variables were summarised with means, standard deviations, or median and interquartile range (IQR) depending on distribution. Linear mixed model analyses were used to assess the amount of change in outcomes in the two groups over the intervention phases (baseline to post‐intervention and post 3-months). The differences in scores between the two treatment groups at 3-months were adjusted for baseline characteristics and stratification factors (age, gender). All tests were two-sided. Within‐group effect sizes for the surveys were calculated using Cohen’s d (d = (M1−M2)/SDpooled, where M1 = baseline mean, M2 = post 3-months mean, and SDpooled = the pooled standard deviation of baseline SD across groups).

## 3 Results

### 3.1 Sample characteristics

Baseline characteristics of participants (n = 81) are categorised into two groups: mindful music (n = 40) and music alone (n = 41); demographics include age, gender, ethnicity, certificate of VI, number of years living with VI, specific types of VI, employment status, and previous mental health history. Key findings indicate a diverse participant profile, with the average age 63 (SD: 19.85), majority of participants being retired, reporting poor mental health, and being severely sight impaired. See **Table 1** for a full breakdown of all demographics. **Table 2** presents music preferences among participants, it includes preferred music listening gadgets/devices, platforms, and uplifting music genres rated by the individual participants. Findings reveal a preference for Alexa/Google Nest/Amazon Echo, with Spotify being the most favoured music platform. Additionally, a majority of participants selected multiple genres.

**Table 1:** Baseline characteristics (mean (SD)/median (IQR) or counts (%)).

	Total N = 81	Mindful music N = 40	Music alone N = 41
Age (years)	63.10 (19.85)	62.56 (14.67)	63.67 (3.01)
Gender			
Male	38 (46%)	12 (30%)	26 (64%)
Female	41(51%)	28 (70%)	13 (31%)
Other	2 (3%)	0	2 (5%)
Ethnicity			
White (English, Welsh, Scottish, Northern Irish or British)	35 (43%)	14 (35%)	21 (51%)
White Other	17 (20%)	10 (25%)	7 (16%)
Black Other	7 (8%)	5 (12%)	2 (5%)
Black Caribbean	3 (4%)	1 (2%)	2 (5%)
Mixed or multiple ethnic background	4 (4%)	3 (8%)	1 (3%)
Indian	6 (8%)	2 (6%)	4 (10%)
Pakistani	1 (2%)	1 (2%)	0
Chinese	3 (4%)	1 (2%)	2 (5%)
Any other ethnic group	5 (7%)	3 (8%)	2 (5%)
Certificate of VI			
Severely sight impaired	45 (55%)	21 (52%)	24 (51%)
Sight impaired	33 (40%)	17 (42%)	16 (39%)
Not registered VI	3 (5%)	2 (6%)	1 (10%)
Vision Impairment			
Eye-Injury	9 (11%)	7 (16%)	2 (4%)
Macula Disease	5 (6%)	1 (3%)	4 (10%)
Diabetic Retinopathy	7 (8%)	2 (5%)	5 (12%)
Retinitis Pigmentosa	4 (4%)	3 (6%)	1 (2%)
Age-related Macular Degeneration	31 (38%)	11 (29%)	20 (50%)
Glaucoma	10 (12%)	5 (11%)	5 (14%)
Hemianopia	1 (1%)	1 (3%)	0
Keratoconus	1 (1%)	1 (3%)	0
Optic Atrophy	2 (3%)	2 (5%)	0
Birdshot Chorioretinopathy	1 (1%)	1 (3%)	0
Nystagmus	1 (1%)	1 (3%)	0
Stargardt’s disease	1 (1%)	0	1 (2%)
Retinopathy of Prematurity	2 (3%)	1 (3%)	1 (2%)
Other-Brain Injury	3 (5%)	2 (5%)	1 (2%)
Other-Stroke	3 (5%)	2 (5%)	1 (2%)
Employment status			
Full-time employed	5 (6%)	1 (2%)	4 (10%)
Part-time employed	6 (7%)	3 (8%)	3 (7%)
Student	5 (6%)	4 (10%)	1 (2%)
Volunteer	20 (25%)	9 (25%)	29 (28%)
Retired	41(51%)	21 (52%)	20 (48%)
Unemployed	4 (5%)	2 (3%)	2 (5%)
Previous mental health history			
Yes	54 (66%)	25 (62%)	29 (69%)
No	27 (34%)	15 (38%)	29 (31%)

**Table 2:** Music preferences across both groups.

	Total N = 81	Mindful music N = 40	Music alone N = 41
Preferred music listening gadget/device			
Ipad/tablet or equivalent	11 (14%)	9 (23%)	2 (5%)
Alexa/Google Nest/Amazon Echo/or other equivalent:	50 (62%)	22 (55%)	28 (69%)
Desktop/Laptop	10 (12%)	4 (10%)	6 (14%)
Smart phone	10 (12%)	5 (12%)	5 (12%)
Preferred music listening platform			
Spotify	38 (47%)	26 (64%)	29 (32%)
Apple Music	29 (35%)	7 (17%)	22 (49%)
Amazon Music	29 (16%)	7 (17%)	6 (17%)
YouTube	1 (2%)	0 (2%)	1 (2%)
Most voted uplifting music genre			
Classical	3 (4%)	1 (2%)	2 (4%)
Jazz	2 (2%)	1 (2%)	1 (2%)
Big Band/Swing	2 (2%)	1 (2%)	1 (2%)
Blues	1 (1%)	0	1 (2%)
Spiritual/Religious:	1 (1%)	0	1 (2%)
Instrumental	3 (4%)	0	3 (9%)
Cultural/ethnic	2 (2%)	1 (2%)	1 (2%)
Country	1 (1%)	1 (2%)	0
Folk	0	0	0
Musical Theatre	2 (2%)	1 (2%)	1 (2%)
Top 40/What’s on the radio	2 (2%)	1 (2%)	1 (2%)
Multiple genres*	62 (79%)	34 (86%)	29 (73%)

*Multiple genres refer to selecting more than one genre from the above list, these also included Band bass, Electronic, Hip-Hop and Rock and Roll.

### 3.2 Feasibility outcomes

#### 3.2.1 Feasibility of recruitment

**Figure 2** illustrates the recruitment process for this study, using a Consort flow diagram. Reasons for not participating in the study included:

- declined to participate (n = 11),
- did not meet eligibility criteria based on the minimum score on screening questionnaire, DASS-21 (n = 9) for depression and/or anxiety and/or stress,
- no access or support to use a music streaming platform at home (n = 6),
- did not have an acquired VI (n = 2).

Eighty-four individuals completed the baseline assessments. The recruitment was conducted on an on-going basis during the eight-month recruitment phase, the average rate of recruitment was 2.6 participants per week over 32 weeks.

#### 3.2.2 Retention

Sixty-nine of the 81 (85%) participants were completed the intervention treatment. Between the intervention phase and post-3-months intervention endpoint, there were dropouts (n = 23), in the mindful music group (*n* = 13) and the music alone group (*n* = 10). One participant in each group lost interest, time constraints led to *n* = 2 withdrawals in each group. Health issues caused *n* =1 dropout in the mindful music group but not in the music alone group. Additionally, *n* = 9 participants in the mindful music group and *n* = 7 in the music alone group were lost due to contact issues, therefore. No reason was provided for dropping out of the study. Therefore, n = 58 (72%) participants remained in the study until the 3-months endpoint. Data from individuals who dropped out were excluded from the statistical analysis of the well-being outcomes (HADS and PSS14) pre/post scores.

#### 3.2.3 Intervention feasibility, acceptability and accessibility

Sixty-two participants (89%: *n* = 30-mindful music, *n* = 32-music alone) rated the intervention as very accessible or accessible and 66 (95%) participants rated very likely or likely to recommend the intervention to a friend (**Table 3**). Fifty-three (77%) out of *n* = 69 participants (*n* = 25-mindful music, *n* = 28-music alone) gave the intervention a satisfaction rating of 7 or higher and *n* = 60 (86%: *n* = 27-mindful music, *n* = 33-music alone) participants rated 7 or higher on how enjoyable they found the intervention (**Table 3**).

**Table 3:** Median (Inter quartile range) intervention feasibility and satisfaction ratings by group post intervention phase.

	Overall N = 69	Mindful music N = 34	Music alone N = 35
How accessible was the intervention?	4 (4,5)	4 (4,5)	4 (4,5)
How satisfied with this intervention?	8 (8, 9)	9 (8,10)	8 (7, 9)
Rate how much you enjoyed completing this intervention?	8 (7, 9)	9 (8, 10)	8 (7, 9)
Rate how likely you are to recommend this intervention to a friend?	8 (7, 9)	8.5 (8, 10)	8 (7,9)
How satisfied with the daily logs? *	7 (6, 8)	**7.5 (7, 8)**	6.5 (6, 7)

Rating scale scores: 5 (very)-and 1 (not at all) or 10 (very)–1 (not at all), with higher scores being more favourable.

*This rating is not representative of the entire study sample as daily logs were completed by (N=11).

#### 3.2.4 Intervention adherence and fidelity

Eleven out of *n* = 69 participants (16%) completed the daily logs *n* = 4 from the mindful music group and *n* = 7 from the music alone group. Of these 11 participants, 10 completed their logs online and 1 recorded them on paper. For the satisfaction ratings of the daily logs, see **Table 3**. Among those who completed their logs online, adherence to the music playlist was 90% (*n* = 10), adherence to the mindful listening instructions audio (mindful music group only) was 100% (*n* = 4), and 50% (*n* = 2) reported that from week three onwards they listened to their mindful music without any audio narrative.

#### 3.2.5 Playlist compilation

A total of *n* = 81 personalised music playlists were prepared based on the information provided in the music preference survey. Additionally, up to two supplementary telephone consultations, each lasting one hour, were conducted when needed which further informed the playlist choices. Twenty-one music consultation were conducted. Music algorithms on the streaming platform, powered by artificial intelligence (AI), was used to analyse the preferences indicated in the surveys and consultations. These algorithms then curated personalised playlists by selecting songs, albums, artists, and genres according to the provided preferences. For more details on the survey section used in playlist compilation, please refer to the **Supplementary Data Sheet 1**.

#### 3.2.6 Daily log analysis

Over the 20 music listening sessions conducted over 4 weeks, only *n* = 11 out of 69 (16%) participants consistently completed the daily logs. Among these, 1 participant listened to their music with another person every time, while 3 participants occasionally listened with someone else. More than 70% (*n* = 9) of these participants engaged in physical movement to the music, such as swaying, clapping, or tapping their feet. One participant noted in the daily log that a particular song reminded them of their ex-husband; however, this memory recall did not lead to any adverse reaction and highlights how music can evoke past memories (27–29). Additionally, *n* = 3 participants reported changes in their routine, including attending a medical appointment, going on a staycation, or experiencing a change in their blood pressure medication dosage. The average duration of music listening for both groups combined, was up to one-hour/daily and the most common listening time was afternoon (12-5pm) closely followed by morning (8am-12pm), refer to **Supplementary Data Sheet 2**.

The median mood rating before music listening for the *n* = 11 participants was 3 (neutral), increasing to 4 (calm) after listening to music. For the mindful music group (*n* = 4), the median mood rating before music listening was 3 (neutral), which improved to 4 (calm) after listening. In the music alone group (*n* = 7), the median mood rating before music listening was 3 (neutral), and it increased to 3.5 (slightly calm) after music listening.

### 3.3 Analysis of psychological assessment data

For the psychological assessments, we analysed the differences in depression (HADS-D), anxiety (HADS-A), and stress (PSS-14) levels between those who completed pre and post evaluations (*n*= 69) and those who completed evaluations at a three-month follow-up (*n*=58) using linear mixed models (**Table 4**). The pre-post results showed a reduction in depression, anxiety and stress scores. However, these improvements were observed to diminish after three months, indicating that the effects of the treatment wear off over time. There were no significant differences between the mindful music and music alone groups in terms of depression and anxiety scores (HADS), however, we identified a significant difference in scores in perceived stress (PSS-14) in favour of mindful music listening.

**Table 4:** Linear mixed models analysis of HADS-A, HADS-D, and PSS-14 at post and 3 months.

Outcomes	Group 1			Group 2			Between group difference
	N	Mean change from baseline (95%CI)	Mean change from post to 3 months (95%CI)	N	Mean change from baseline (95%CI)	Mean change from post to 3 months (95%CI)	Mean difference (95%CI)
**HADS-A**	41			40			
post	34	-5.41 (-6.25, -4.56)	–	35	-3.89 (-4.72, -3.05)	–	0.80 (-0.68, 2.29)
3-month	29	-2.10 (-2.99, -1.21)	3.31 (2.40, 4.21)	29	-1.17 (-2.07, -0.28)	2.71 (1.81,3.62)	0.21 (-1.34, 1.76)
**HADS-D**	41			40			
post	34	-5.11 (-5.94,-4.28)	–	35	-4.53 (-5.36,-3.71)	–	0.86 (-0.58, 2.29)
3-month	29	-0.92 (-1.79, -0.05)	4.19 (3.31,5.07)	29	-1.95 (-2.83,-1.07)	2.58 (1.69,3.47)	-0.75 (-2.24, 0.75)
**PSS-14**	41			40			
post	34	-9.96 (-11.65,-8.27)	–	35	-6.83 (-8.51,-5.15)	–	3.24 (0.82,5.66)
3-month	29	-5.53 (-7.31,-3.74)	4.43 (2.61,6.26)	29	-2.78 (-4.58,-0.98)	4.05 (2.23,5.87)	2.86 (0.27,5.44)

## 4 Discussion

The intervention protocol is feasible but requires modifications for a future efficacy trial due to unknown treatment fidelity stemming from low completion of daily logs (16%). Reasons for these included burden of care, time constraints, completion of logs not being emphasised as mandatory. In future trials, it is essential to stress the importance of completing the daily logs as a requirement of participating in the trial. Additionally, alternative methods such as audio diaries or shorter, more user-friendly logging processes should be considered to improve adherence.

While the average enrolment rate (70% of those invited) and retention rate (85% of those recruited) suggest feasible recruitment methods, the non-compliance in completing the daily logs hinders data analysis when considering the daily impact of the music listening intervention across the recruited participant population. In the end of intervention survey participants rated the intervention highly with regard to accessibility to the music playlist, satisfaction and enjoyment (**Table 3**). The outcome measures indicate treatment related effects, with reductions in depression, anxiety, and stress levels. No significant differences were found between the MML and ML groups in depression and anxiety scores, with both indicating significant reductions (**Table 4**), with a notable distinction in perceived stress scores favouring MML.

### 4.1 Adherence, retention and attrition

Good listening adherence was noteworthy in both music groups, aligning with Baylan et al. (2020), who also reported good adherence in the music group (58). Both treatment arms in this study exhibited attrition rates that surpassed expectations for psychological therapies, consistent with a systematic review and two meta-analyses encompassing 104 randomised controlled trials (48).

It is plausible that adherence and retention outcomes in a music intervention study can be influenced by the element of personalised music. Personalised music has been recognised as a potentially impactful factor in enhancing engagement and participation in music interventions. This is supported by several studies and interventions exploring the benefits of personalised music in healthcare settings, such as in dementia care, mental health, and rehabilitation (63–66). The idea of tailoring music choices to individuals’ preferences can create a more meaningful experience (66), and promote a sense of ownership, relevance, and enjoyment, thereby increasing motivation to adhere to the intervention and stay involved.

### 4.2 Treatment fidelity

Collecting daily log data is an underused method in health research (67), because the daily commitment to data entry can be demanding for participants, resulting in low participation and incomplete data (67). Additionally, handling and analysing the large amount of data from daily logs can be difficult for researchers, especially without advanced tools (68). Consequently, researchers may omit the use of such data collection, missing insightful details that daily logs can provide on individual’s experiences of health behaviour and wellbeing (69). To address fidelity in future studies, potential strategies might include audio logs for oral self-recording of responses, more frequent reminders, and incentives to illustrate to participants the value of the data they provide (67).

### 4.3 Follow-up

The three-month follow-up phase indicated that when the treatment stops, the effects are not sustained. This pattern aligns with findings from several meta-analyses on the impact of music listening on anxiety, where the effects were positive during the treatment phase but declined at follow-up (48, 64, 68, 69). Notably, the literature highlighted only a limited number of studies reported the duration of the follow-up period (post-intervention), with inconsistent reporting, indicating a need for further research to explore the methods of extending the effects of the intervention (64, 68, 69).

### 4.4 Sensitivity of outcome measures

The Hospital Anxiety and Depression Scale (HADS) and the Perceived Stress Scale (PSS) are regarded as sensitive measures for assessing mental health outcomes in populations, including those with VI (55). Both scales feature items that are relevant and relatable to VI individuals, enhancing participant engagement and response accuracy. Extensive research supports the validity and reliability of the HADS and PSS across various populations, affirming their effectiveness in capturing psychological constructs (70, 71). Moreover, their ease of administration and widespread use in research with visually impaired populations further underscore their utility and credibility (55, 61). Overall, the HADS and PSS offer valuable tools for comprehensively assessing mental health outcomes in VI individuals, facilitating a nuanced understanding of their psychological well-being (55, 61, 72).

### 4.5 Study limitations and future directions

The first limitation of this study was the recruitment radius, although recruitment for this music intervention was feasible, it was restricted to the researcher’s local areas, hence potential participants outside of the local recruitment map were excluded. Conducting a multisite pilot would be essential to evaluate the trial’s effectiveness across a more geographically diverse and representative sample to minimise single-site bias.

While music listening had a positive impact on well-being, as indicated by the high rankings on the post-intervention survey, we were unable to collect all the planned data, such as adherence and fidelity. Participants faced difficulties accessing the daily logs on the survey platform due to inconvenience, time constraints, or lack of support using the platform on their electronic devices. In contrast, listening to music was a simple task—just pressing play or using voice commands on a smart device—while daily logging on the survey platform required extra steps. This likely contributed to the low data collection rate, especially since completing the daily logs was optional. Future studies should use an audio diary with voice command assistance, as well as make the daily logs mandatory to ensure we capture all data.

AI-generated playlists commonly found on streaming platforms offer personalised recommendations tailored to users’ preferences and listening habits (51, 73, 74). However, this study did not obtain feedback on the playlist generation method, which could provide useful insights into participants’ experience of this and whether there were other routes to establishing the most effective playlists to meet the intervention aims. Although participants’ feedback implied that their personalised playlists were enjoyable (**Table 3**), the survey questions did not exclusively ask them about the compilation process, their experience of it, and explore alternatives. Evaluating the usefulness of the streaming platform algorithm that was used in producing the playlists was beyond the scope of this research. The platform algorithm was merely a tool to efficiently generate a large playlist that the researcher felt would be sufficient (number of tracks) for the duration of the intervention. Hence, this is an area that requires more research as this may have consequences for both treatment adherence and efficacy.

Another limitation of this study is that it does not assess participant satisfaction with the playlist production process, including feedback on AI-based playlist generation methods, nor does it explore alternative approaches such as manual playlist curation for comparison. Future studies could address this gap by investigating participant satisfaction across different playlist production processes, which could ensure that playlists effectively minimise potential negative emotional triggers while achieving therapeutic objectives. Moreover, comparing AI-based methods with manual curation could provide valuable insights into user preferences and help optimise personalisation to enhance user experience and engagement.

The psychological outcome measures in this study relied on self-reports, which were collected remotely, lacking objectivity and potentially leading to inconsistent data (75–77). Moreover, perceived and experienced emotions may differ due to a lack of emotional connection online (76). Additionally, physiological outcome measures offer objectivity and quantifiable data, enabling measurable changes (75–78) and correlations with biological processes such as stress, which are crucial in medical research (75, 77). A future trial could benefit from including physiological stress measures, such as heart rate and cortisol levels, alongside behavioural observations (78).

As the researcher built a rapport with the participants, there is a risk that they were overly positive regarding the intervention outcomes (79). This could be improved by implementing additional measures to ensure objectivity, such as incorporating independent assessors or utilising blinded evaluations in future studies.

## 5 Conclusion

Recruitment and retention, and collection of psychological outcome data indicate that an efficacy study is feasible but with modification. In order to fully understand any treatment related effects more daily listening log data is needed, including whether participants listen to their study playlists or other material, if they follow the mindfulness protocol, if they listen with anyone, when and for what duration. A review of relevant literature and patient, carer and public involvement data from those living with an acquired visual impairment on methods and frequency of preferred daily listening log data collection would be necessary, after which a small pilot study would be required to test them. Determining whether algorithmically driven playlist compilation is appropriate or more personally tailored ones, which might avoid any music that the listener has negative associations with, would also improve a future study. Another consideration for further feasibility work would be objective measures of stress and anxiety.

## Data Availability

The raw data supporting the conclusions of this article will be made available by the authors, without undue reservation.
