# Supplementary material for: Music listening for psychological well-being in adults with acquired vision impairment: a feasibility randomised controlled trial
Source: Front Psychiatry. 2025 Feb 25;16:1505283. doi: 10.3389/fpsyt.2025.1505283 (PMC11893566; doi:10.3389/fpsyt.2025.1505283)
Supplement: Supplementary file 1 [file DataSheet1.pdf]

Music Preference Questionnaire

The questionnaire for the selection of music was adapted from the APMP (The Assessment of Personal Music Preference) devised by Gerdner et al. (2000)

It includes questions relating to participants' musical background and their preferred genres, composers, and performers.

Instructions

- Please take as long as necessary to complete this survey.
- It is **not** compulsory to complete in one sitting, you may return to it after a couple of hours or days to give you time to have a think about your responses.
- It may be a good idea to complete this with someone else close to you to help you think back to all the songs and artist you enjoy.
- Once you've completed this survey, the researcher Nurbanu Somani may get in touch with you to arrange a telephone/Teams/Zoom consultation if your survey responses are not detailed enough for her to create a long enough personalised playlist for the duration of the 4-weeks intervention. The purpose of the consultation will be to clarify and assess your responses to potential musical pieces that can be added to your personalised music listening playlist. The consultation may last up to an hour.

1. What types of music do you enjoy?

Please select all that apply:

Country and Western
Classical
Spiritual/Religious
Big Band/Swing
Folk
Blues
Jazz
Rock and Roll
Hip Hop
K-Pop

Rap
Electronic
Easy Listening

Cultural or Ethnic (examples: Czech polkas, Ravi Shankar Indian sitar)

New Age
Musicals
Pop
Opera
Choral
Military
Brass Band

Any other

2. Do/did you play a musical instrument? *Yes /No*

If yes, what do/did you play?

3. Do you have any positive memories playing the instrument?

No/Yes

4. If yes, are there any particular pieces you enjoy playing or have played in the past or something that you would like to play in the future?

5. Do/did you enjoy singing? *Yes/ No*

If yes, what type of songs do you enjoy singing?

6. Do you listen to music on the radio?

Yes/ No

If yes, what radio station/s do you listen to?

7. Do you like to dance?

Yes/ No

If yes, what kind of music do/did you like to dance to?

8. Do you like to listen to singing or instrumental music or both?

9. Are there any instruments that you particularly like to listen to?

Singing
Piano
Keyboard
Violin
Cello
Brass
Harp
Flute

Oboe

Clarinet
Acoustic Guitar
Electric Guitar
Bass Guitar
Bagpipes
World instruments
Accordion
Organ
Drums

Other

10. Are there any instruments that you don’t enjoy listening to?

11. Can you remember buying your first record or streaming your first song? No/Yes

Do you remember what it was?

12. Are there any particular performers, singers or bands that you enjoy listening to?

13. Do you have any favourite composers, pieces or songs?

14. Can you think of any music that makes you feel happy?

15. Can you think of any music that makes you relax?

16. Can you think of any music that you find particularly moving?

17. Is there any music that reminds you of a particular place or event?

18. Would you prefer to listen to short pieces or longer pieces?

19. Would you prefer to listen to lively upbeat music or something slower and more relaxed?

20. Can you identify any music that you would like to exclude that makes you feel sad or low, possibly reminding you of a sad event or difficult period in your life?

21. Type below the titles of the songs, singers/composers/artists of the music, music genres, albums, or playlist. **(This information is very important for this study so please give as much as detail as possible)**

22. Are there types of music that you don't enjoy?
