# Supplementary material for: Music listening for psychological well-being in adults with acquired vision impairment: a feasibility randomised controlled trial
Source: Front Psychiatry. 2025 Feb 25;16:1505283. doi: 10.3389/fpsyt.2025.1505283 (PMC11893566; doi:10.3389/fpsyt.2025.1505283)
Supplement: Supplementary file 3 [file DataSheet3.pdf]

Median (IQR) daily log rating over the 20 days intervention

	Overall<br>*N = 11*	Mindful music<br>*N = 4*	Music alone<br>*N = 7*
Q1. Time Of day	3 (2, 4)	3 (2, 4)	3 (2, 4)
Q2. How long did you listen to music for	2.5 (2, 3)	2 (2, 3)	3 (2, 3)
Q3. Did you move along to the music	1 (1, 1)	1 (1, 1.25)	1 (1, 1)
Q4. Did you listen to what was on the playlist	1 (1, 1)	1 (1, 1)	1 (1, 1)
Q5. Did you listen to the music alone?	1 (1, 1)	1 (1, 1)	(1, 1)
Q6. Have you changed anything in your routine today? (For example: any new medications taken)*	2 (2, 2)	2 (2, 2)	2 (2, 2)
Q7. Did you listen to the music mindfully with the recorded narrative or on your own without it.	N/A	1 (1, 2)	N/A

Note: Rating scale scores:
Q1 (1 = Early Morning, 2 = Morning, 3 = Early Afternoon, 4 = Afternoon, 5 = Evening, 6 = Late Evening)
Q2 (1 = Less than 20 Minutes, 2 = 20-40 Minutes, 3 = 40 Minutes-1 Hour, 4 = More than 1 Hour)
Q3-Q5 (1 = Yes, 2 = No)
Q6 (1 = Recorded narrative, 2 = Own your own with the recorded narrative)
* Doctor prescribed bisoprolol to a participant on day 4, Music listening time changed because of hospital appointment, music listening time changed, BP tablet dosage changed on day 10, Participant started taking Naproxen on day 13.

Additional collected data: Please tell me if you have had any adverse reactions to your listening experience today. Use this space to make notes: (For example (i) were there any songs that made you upset or caused distress and state the reasons why and (ii) any other factors you feel may have influenced your music listening experience?)

Day 6- one participant reported a song reminded her of her ex husband
