# Supplementary material for: Music listening for psychological well-being in adults with acquired vision impairment: a feasibility randomised controlled trial
Source: Front Psychiatry. 2025 Feb 25;16:1505283. doi: 10.3389/fpsyt.2025.1505283 (PMC11893566; doi:10.3389/fpsyt.2025.1505283)
Supplement: Supplementary file 4 [file DataSheet4.docx]

# References

1. Rafaely, L., Carmel, S., & Bachner, Y. G. Subjective well-being of visually impaired older adults living in the community. *Aging Ment Health.* (2018) 22:1229-1236. doi:[10.1080/13607863.2017.1341469](https://doi.org/10.1080/13607863.2017.1341469)
2. Park, H. Y., Chong, H. J., & Kim, S. J. A comparative study on the attitudes and uses of music by adults with visual impairments and those who are sighted. *J Vis Impair Blind*. (2015) 109:303-316. doi: [10.1177/0145482X1510900406](https://doi.org/10.1177/0145482X1510900406)
3. Naylor, P. D., & Labbé, E. E. Exploring the effects of group therapy for the visually impaired. *Br J Vis Impair.* (2017) 35:18-28. doi: 10.1177/0264619616671976
4. Robb, S.L. Music Interventions and Group Participation Skills of Preschoolers with Visual Impairments: Raising Questions about Music, Arousal, and Attention, *Journal of Music Therapy* (2003), 40:4: 266–282. [doi: 10.1093/jmt/40.4.266](https://doi.org/10.1093/jmt/40.4.266)
5. Horowitz, A., Reinhardt, J. P., & Kennedy, G. J. Major and subthreshold depression among older adults seeking vision rehabilitation services. *Am J Geriatr Psychiatry.* (2005) 13:180–187. doi: 10.1176/appi.ajgp.13.3.180
6. Rees, G., Tee, H. W., Marella, M., Fenwick, E., Dirani, M., & Lamoureux, E. L. Vision-specific distress and depressive symptoms in people with vision impairment. *Invest Ophthalmol Vis Sci*. (2010) 51:2891–2896. doi: 10.1167/iovs.09-5080
7. Nyman, S. R., Dibb, B., Victor, C. R., & Gosney, M. A. Emotional well-being and adjustment to vision loss in later life: A meta-synthesis of qualitative studies. *Disabil Rehabil*. (2012) 34: 971-981. doi: 10.3109/09638288.2011.626487
8. Brunes, A., & Heir, T. Social interactions, experiences with adverse life events and depressive symptoms in individuals with visual impairment: A cross-sectional study. *BMC Psychiatry* (2020). 20:1-8. doi: 10.1186/s12888-020-02652-7
9. Virgili, G., Parravano, M., Petri, D., Maurutto, E., Menchini, F., Lanzetta, P., et al. The association between vision impairment and depression: a systematic review of population-based studies. *J. Clin. Med* (2022) 11:2412. doi: 10.3390/jcm11092412
10. Evans, J. R., Fletcher, A. E., & Wormald, R. P. Depression and anxiety in visually impaired older people. *J.Ophthalmol.* (2007) 114:283–288. doi:  [10.1016/j.ophtha.2006.10.006](https://doi.org/10.1016/j.ophtha.2006.10.006)
11. Augustin, A., Sahel, J. A., Bandello, F., Dardennes, R., Maurel, F., Negrini, C., et al. Anxiety and depression prevalence rates in age-related macular degeneration. Invest *Ophthalmol Vis Sci* (2007). 48:1498–1503. doi: 10.1167/iovs.06-0761
12. Van der Aa, H. P., Comijs, H. C., Penninx, B. W., van Rens, G. H., & van Nispen, R. M. Major depressive and anxiety disorders in visually impaired older adults. *Invest Ophthalmol Vis Sci.* (2015) 56:849–854. doi: [10.1167/iovs.14-15848](https://doi.org/10.1167/iovs.14-15848)
13. Van Munster, E. P., Van der Aa, H. P., Verstraten, P., & Van Nispen, R. M. Barriers and facilitators to recognize and discuss depression and anxiety experienced by adults with vision impairment or blindness: a qualitative study. *BMC Health Serv Res.* (2021) 21:749. doi: 10.1186/s12913-021-06682-z
14. Burmedi, D., Becker, S., Heyl, V., Wahl, H. W., & Himmelsbach, I. Emotional and social consequences of age-related low vision. *Vis. Impair. Res.* (2002) 4:47-71. doi: 10.1076/vimr.4.1.47.15634
15. Rabiee, P., Mann, R., Birks, Y., & Wilberforce, M. The experience of loneliness and living with sight loss in English care homes. *J. Aging Stud* (2021) 57:1009-13. doi: 10.1016/j.jaging.2021.100913
16. Hodge, S. & Eccles, F. Loneliness, social isolation and sight loss: *A literature review conducted for Thomas Pocklington Trust.* Division of Health Research: Lancaster University. (2013). Retrieved from: <https://eprints.lancs.ac.uk/id/eprint/68597/1/loneliness_social_isolation_and_sight_loss_final_report_dec_13.pdf>
17. Rokach, A., Berman, D., & Rose, A. Loneliness of the Blind and the Visually Impaired. *Front. Psychol*. (2021) 12:641-711. doi:10.3389/fpsyg.2021.641711
18. Woodward, K*. Psychosocial studies: An introduction*: Routledge, an imprint of Taylor & Francis. (2015).
19. Diener, E. Subjective well-being: The science of happiness and a proposal for a national index. *Am Psychol.* (2000) 55:34. doi: 10.1037/0003-066X.55.1.34?
20. Ryff, C. D., & Keyes, C. L. M. The structure of psychological well-being revisited. *J Pers Soc Psychol.* (1995) 69:719. doi: 10.1037/0022-3514.69.4.719
21. Sweeting, J., Merom, D., Astuti, P.A.S., Antoun, M., Edwards, K., Ding, D. Physical activity interventions for adults who are visually impaired: a systematic review and meta-analysis. *BMJ Open*. (2020) 10:e034036. doi: 10.1136/bmjopen-2019-034036.
22. Levens, M. Art therapy with the visually impaired. *Insight* (1986). 4:21-23. [doi: 10.1177/026461968600400107](https://doi.org/10.1177/026461968600400107)
23. Chan, S.H.W., Cheung, M.Y.C., Chiu, A.T.S., Leung, M.H.T., Kuo, M.C.C., Yip, D.Y.C., et al. Clinical effectiveness of mindfulness-based music therapy on improving emotional regulation in blind older women: A randomized controlled trial*. Integr Med Res* (2023) 12:100993. doi: 10.1016/j.imr.2023.10099
24. Somani, N., Beukes, E., Street, A., Lindsay, R., & Allen, P. M. Music-based interventions to address well-being in people with a vision impairment: protocol for a scoping review. *BMJ open.* (2022) 12:e054268. doi: 10.1136/bmjopen-2021-054268
25. de la Torre-Luque, A., Caparros-Gonzalez, R. A., Bastard, T., Vico, F. J., & Buela-Casal, G. Acute stress recovery through listening to Melomics relaxing music: A randomized controlled trial. *Nordic Journal of Music Therapy* (2016) 26:124–141. doi: 10.1080/08098131.2015.1131186
26. Radstaak, M., Geurts, S.A.E.,Brosschot, J.F., Kompier, M.A.J. Music and Psychophysiological Recovery from Stress. *Psyc Med* (2014) 76:529-537. doi:10.1097/PSY.0000000000000094
27. Jäncke, L. Music, memory and emotion*. J Biol* (2008) 7:21. doi: 10.1186/jbiol82.
28. Baylan, S., McGinlay, M., MacDonald, M., Easto, J., Cullen, B., Haig, C., et al. Participants’ experiences of music, mindful music, and audiobook listening interventions for people recovering from stroke. *Ann N Y Acad Sci.* (2018) 1423:349-59. doi: 10.1111/nyas.13618
29. Fredrickson, B. L. The broaden–and–build theory of positive emotions. Philosophical Transactions of the Royal Society of London.Series B: *Biol. Sci*. (2004) 359:1367-77. doi: 10.1098/rstb.2004.1512
30. Seligman, M. E. *Flourish: A visionary new understanding of happiness and well-being*. Simon and Schuster (2011).
31. Somani, N., Beukes, E., Street, A., Lindsay, R., Smith, L., & Allen, P. M. Effectiveness of music-based interventions to address well-being in people with a vision impairment: a scoping review. *BMJ open.* (2023) 13:e067502. doi: 10.1136/bmjopen-2021-067502
32. Linnemann, A., Ditzen, B., Strahler, J., Doerr, J. M., & Nater, U. M. Music listening as a means of stress reduction in daily life. *Psychoneuroendocrinology.* (2015) 60: 82-90. doi: 10.1016/j.psyneuen.2015.06.008
33. Orem, D. E. *Nursing: Concepts of practice*. McGraw-Hill New York (1971).
34. Skånland, M.S. *'A technology of well-being: A qualitative study on the use of MP3 players as a medium for musical self-care'*, Norwegian Academy of Music (2012).
35. Sung, H.C., Chang, A.M., and Lee, W.L. 'A preferred music listening intervention to reduce anxiety in older adults with dementia in nursing homes'. *J. Clin. Nurs* (2010) 19:1056-1064. doi: 10.1111/j.1365-2702.2009.03016.x
36. Raglio, A. Therapeutic music listening as telehealth intervention. *Complement Ther Clin Pract.* (2020) 41:1012-45. doi: 10.1016/j.ctcp.2020.101245
37. Vaudreuil, R., Langston, D.G., Magee, WL., Betts, D., Kass, S., and Levy, C. Implementing music therapy through telehealth: considerations for military populations. Assistive Technology. *Disabil. Rehabil*. (2022) 17:201-10. doi:10.1080/17483107.2020.1775312
38. Awaludin, S., Nurachmah, E., Soetisna, T.W., and Umar, J. The effect of a smartphone-based perioperative nursing intervention: Prayer, education, exercise therapy, hypnosis, and music toward pain, anxiety, and early mobilization on cardiac surgery. *J. Public Health Res.* (2022) 11:2021. doi.10.4081/jphr.2021.2742
39. Sorkpor, S.K., Miao, H., Moore, C., Johnson, C.M., Santa Maria, D.M., Pollonini, L., and Ahn, H. Listening to Remotely Monitored Home-based Preferred Music for Pain in Older Black Adults with Low Back Pain: A Pilot Study of Feasibility and Acceptability. *J. Pain Manag*. (2023) 24:e102-e108. doi: 10.1016/j.pmn.2023.07.001
40. 40. Barker I, Steventon A, Williamson R. Deeny, SR. Self-management capability in patients with long-term conditions is associated with reduced healthcare utilization across a whole health economy: cross-sectional analysis of electronic health records. BMJ Qual. Saf. (2018) 27:989–99. doi: 10.1136/bmjqs-2017-007635
41. Du Feu, M., Fergusson, K. Sensory impairment and mental health. *Adv Psychiatr Treat* (2003) 9:95-103. doi: 10.1192/apt.9.2.95
42. Nyman, S.R., Gosney, M.A., Victor, C.R. Psychosocial impact of visual impairment in working-age adults. *Br J Ophthalmol* (2010) 94:1427-31. doi: 10.1136/bjo.2009.164814
43. Eldridge, S.M., Chan, C.L., Campbell, M.J., Bond, C.M., Hopewell, S., Thabane, L., et al. CONSORT 2010 statement: extension to randomized pilot and feasibility trials. *BMJ* (2016) 355:i5239. doi: 10.1136/bmj.i5239
44. Royal National Institute of Blind People (RNIB). *The criteria for certification*. Available from: [https://www.rnib.org.uk/your-eyes/navigating-sight-loss/registering-as-sight-impaired/the-criteria-for-certification/?](https://www.rnib.org.uk/your-eyes/navigating-sight-loss/registering-as-sight-impaired/the-criteria-for-certification/?utm_source=chatgpt.com) [Accessed 10/10/2024]
45. Snellen, H. *Probebuchstaben zur bestimmung der sehschärfe*. Verlag von Hermann Peters (1873)
46. Crawford, J.R., & Henry, J.D. The Depression Anxiety Stress Scales (DASS): Normative data and latent structure in a large non‐clinical sample. *Br. J. Clin. Psychol.* (2003) 42:111-131. doi: 10.1348/014466503321903544
47. Faul, F., Erdfelder, E., Lang, A., & Buchner, A. G* Power 3: A flexible statistical power analysis program for the social, behavioral, and biomedical sciences. *Behav. Res. Methods.* (2007) 39:175-191. doi: 10.3758/bf03193146
48. de Witte, M., Spruit, A., van Hooren, S., Moonen, X., & Stams, G. Effects of music interventions on stress-related outcomes: a systematic review and two meta-analyses'. *Health Psychol. Rev.* (2020) 14:294-324. doi: 10.1080/17437199.2019.1627897
49. Vickers, A.J. How to randomize. *J. Integr. Oncol*. (2006) 4:194. doi: 10.2310/7200.2006.023
50. Gerdner, L.A. Individualized music intervention protocol. *J. Gerontol. Nurs.* (1999) 25:10-16. doi: 10.3928/0098-9134-19991001-08
51. Freeman, S., Gibbs, M., & Nansen, B. Don’t mess with my algorithm: Exploring the relationship between listeners and automated curation and recommendation on music streaming services. *First Monday* (2022). doi: 10.5210/fm.v27i1.11783
52. Segal, Z., Williams, M., & Teasdale, J. *Mindfulness-based cognitive therapy for depression*. Guilford Publications, New York, USA (2018).
53. Hoffmann, T.C., Glasziou, P.P., Boutron, I., Milne, R., Perera, R., Moher, D., Altman, D.G., Barbour, V., Macdonald, H., Johnston, M., *et al*. Better reporting of interventions: template for intervention description and replication (TIDieR) checklist and guide. *BMJ* (2014) 348:1687. doi:10.1136/bmj.g1687.
54. Lotery A, Xiao Xu, Zlatava G, Loftus, J. Burden of illness, visual impairment and health resource utilisation of patients with neovascular age-related macular degeneration: results from the UK cohort of a five-country cross-sectional study. Br J Ophthalmol. (2007) . 91:1303–7. doi: 10.1136/bjo.2007.116939Demmin, D.L., & Silverstein, S.M. Visual Impairment and Mental Health: Unmet Needs and Treatment Options. *Clin Ophthalmol* (2020) 14:4229. doi: 10.2147/OPTH.S258783
55. Zigmond, A.S., & Snaith, R.P. The hospital anxiety and depression scale. *Acta Psychiatr. Scand*. (1983) 67:361-370. doi: 10.1111/j.1600-0447.1983.tb09716.x
56. Cohen, S., Kamarck, T., & Mermelstein, R. *Perceived stress scale*. Measuring Stress: A Guide for Health and Social Scientists (1994) 10:1-2.
57. Baylan, S., Haig, C., MacDonald, M., Stiles, C., Easto, J., Thomson, M., Cullen, B., Quinn, T.J., Stott, D., Mercer, S.W. and Broomfield, N.M. Measuring the effects of listening for leisure on outcome after stroke (MELLO): A pilot randomized controlled trial of mindful music listening', *Int J Stroke* (2020) 15:149-158. doi: 10.1177/1747493019841250
58. Clover K, Lambert SD, Oldmeadow C, Britton B., *et al*. Apples to apples? Comparison of the measurement properties of hospital anxiety and depression-anxiety subscale (HADS-A), depression, anxiety and stress scale-anxiety subscale (DASS-A), and generalised anxiety disorder (GAD-7) scale in an oncology setting using Rasch analysis and diagnostic accuracy statistics. *Curr Psychol.* (2020) 1-10. doi: 10.1007/s12144-020-00906-x
59. Clover K, Lambert SD, Oldmeadow C, Britton B, King M, Mitchell A, et al. Apples to apples? Comparison of the measurement properties of hospital anxiety and depression anxiety subscale (HADS-A), depression, anxiety and stress scale-anxiety subscale (DASS-A), and generalised anxiety disorder (GAD-7) scale in an oncology setting using Rasch analysis and diagnostic accuracy statistics. *Curr Psychol*. (2020) 41:1–10. doi: 10.1007/s12144-020-00906-x
60. Lee E. Review of the psychometric evidence of the perceived stress scale, *Asian Nurs Res (Korean Soc Nurs Sci)* (2012) 6:121-7. doi: 10.1016/j.anr.2012.08.004
61. Soria-Reyes LM, Cerezo MV, Alarcón R, Blanca MJ. Psychometric properties of the perceived stress scale (pss‐10) with breast cancer patients, *Stress Health.* (2023) 39:115-24. doi: 10.1002/smi.3170
62. McManus S, Bebbington P, Jenkins R, Brugha T. (eds.) (2016) Mental health and wellbeing in England: Adult Psychiatric Morbidity Survey 2014. *Leeds: NHS Digital*. Available at: http://content.digital.nhs.uk/catalogue/PUB21748/apms-2014-full-rpt.pdf
63. Harney, C., Johnson, J., Bailes, F. and Havelka, J. Is music listening an effective intervention for reducing anxiety? A systematic review and meta-analysis of controlled studies, *Musicae Scientiae*. (2023) 27:278-298. doi:10.1177/10298649211046979
64. Panteleeva, Y., Ceschi, G., Glowinski, D., Courvoisier, D.S. and Grandjean, D. Music for anxiety? Meta-analysis of anxiety reduction in non-clinical samples. *Psychology of Music*. (2018) 46:473-487. doi: 0.1177/0305735617712424
65. McManus S, Bebbington P, Jenkins R, Brugha T. (eds.) (2016) Mental health and wellbeing in England: Adult Psychiatric Morbidity Survey 2014. *Leeds: NHS Digital*. Available at: http://content.digital.nhs.uk/catalogue/PUB21748/apms-2014-full-rpt.pdf
66. Gaviola, M.A., Inder, K.J., Dilworth, S., Holliday, E.G. and Higgins, I. Impact of individualized music listening intervention on persons with dementia: A systematic review of randomised controlled trials', *Australas J Ageing*. (2020) 39:10-20. doi:10.1111/ajag.12642
67. Gerdner, L.A. *Evidence-based guideline: Individualized music for persons with dementia.* Ann Arbor, Michigan: National Nursing Practice Network (2013).
68. Milligan, C., Bingley, A. and Gatrell, A. Digging deep: Using diary techniques to explore the place of health and well-being amongst older people. *Soc Sci Med* (2005) 61:1882-1892. doi: 10.1016/j.socscimed.2005.04.002
69. Elliott H. The use of diaries in sociological research on health experience*. Sociol. Res. Online* (1997) 2:38-48. doi: doi/10.5153/sro.38
70. Prochaska J.J., Nigg C.R., Spring B., Velicer W.F., Prochaska J.O. The benefits and challenges of multiple health behavior change in research and in practice*. Prev Med.* (2010) 50:26-9. doi: 10.1016/j.ypmed.2009.11.009.
71. Harney, C., Johnson, J., Bailes, F. and Havelka, J. Is music listening an effective intervention for reducing anxiety? A systematic review and meta-analysis of controlled studies, *Musicae Scientiae*. (2023) 27:278-298. doi:10.1177/10298649211046979
72. Panteleeva, Y., Ceschi, G., Glowinski, D., Courvoisier, D.S. and Grandjean, D. Music for anxiety? Meta-analysis of anxiety reduction in non-clinical samples. *Psychology of Music*. (2018) 46:473-487. doi: 0.1177/0305735617712424
73. Bjelland I, Dahl A.A, Haug T. T and Neckelmann D. The validity of the Hospital Anxiety and Depression Scale: an updated literature review. *J. Psychosom. Res.* (2002) 52:69-77. doi:10.1016/S0022-3999(01)00296-3.
74. Lee EH. Review of the psychometric evidence of the perceived stress scale. *Asian Nurs Res (Korean Soc Nurs Sci).* (2012) 6:121-7. doi: 10.1016/j.anr.2012.08.004
75. van der Aa HP, Margrain TH, van Rens GH, Heymans MW, van Nispen RM. Psychosocial interventions to improve mental health in adults with vision impairment: systematic review and meta-analysis. *Ophthalmic Physiol Opt* (2016). 36:584-606. doi: 10.1111/opo.12313.
76. Goh BJ, Soong HC and Ayyasamy, RK. User Song Preferences using Artificial Intelligence. *IEEE International Conference on Computing (ICOCO).* (2021) pp. 330-335. doi: 10.1109/ICOCO53166.2021.9673565*.*
77. Álvarez P, García de Quirós J and Baldassarri SA. Web System Based on Spotify for the automatic generation of affective playlists. *Communications in Computer and Information Science*, Springer. (2021). doi: 10.1504/IJBHR.2011.043414
78. Paulhus DL and Vazire S. *The self-report method. Handbook of research methods in personality psychology*. (2007) pp. 224-239. Guilford Press.
79. Rosenman R, Tennekoon V& Hill LG. Measuring bias in self-reported data. *IJBHR.* (2011) *2*: 320-332.doi: 10.1504/IJBHR.2011.043414
80. Dorsey, A., Scherer, E., Eckhoff, R., & Furberg, R. *Measurement of Human Stress: A Multidimensional Approach*. RTI Press (2022). doi:10.0.3768/rtipress.2022.op.0073.2206
81. Piazza, J. R., Almeida, D. M., Dmitrieva, N. O., & Klein, L. C. Frontiers in the use of biomarkers of health in research on stress and aging. Series B. *J Gerontol B Psychol Sci Soc Sci.* (2010) 65:513–525. doi: 10.1093/geronb/gbq049
82. Horsfall M, Eikelenboom M, Draisma S, Smit JH. The Effect of Rapport on Data Quality in Face-to-Face Interviews: Beneficial or Detrimental?. *Int J Environ Res Public Health* (2021) 8:10858. doi: 10.3390/ijerph182010858.
